# Adaptation to Spanish of the “Relational Needs Satisfaction Scale”: Translation and psychometric testing

**DOI:** 10.3389/fpsyg.2022.992205

**Published:** 2022-08-23

**Authors:** Ioseba Iraurgi, Ignacio Gómez-Marroquín, Richard Erskine, Amaia Mauriz, Silvia Martínez-Rodríguez, Susana Gorbeña, Gregor Žvelc

**Affiliations:** ^1^Faculty of Health Sciences, University of Deusto, Bilbao, Spain; ^2^Institute for Integrative Psychotherapy, Vancouver, BC, Canada; ^3^Instituto Bios de Psicoterapia Integrativa, Bilbao, Spain; ^4^Department of Psychology, Faculty of Arts, University of Ljubljana, Ljubljana, Slovenia

**Keywords:** Relational Needs Satisfaction Scale, test translation, psychometric adequacy, validity evidence, integrative psychological therapy (IPT)

## Abstract

This article aims to adapt to Spanish the Relational Needs Satisfaction Scale (RNSS) and to test the factor structure with a clinical and a non-clinical sample. A total of 459 individuals completed the RNSS, a measure of life satisfaction and of psychological wellbeing. Results showed that the translation was adequate. An exploratory and confirmatory factor analysis was conducted followed by the test of three models that confirmed the five-factor structure and the second-order global factor proposed in the original study, and in adaptations to other languages. The advantages and disadvantages of these models are discussed. Correlations of the RNSS with life satisfaction and psychological wellbeing measures were in the expected direction, providing evidence of convergent validity. The Spanish version of the RNSS is a valid and reliable measure of the construct it was intended to measure, though some improvements in item wording could be incorporated and tested (for instance, item 18 should be positively worded as the rest of the items in order to avoid the effect of negative wording).

## Introduction

Optimal human functioning necessarily requires a relationship with others (Ryan and Deci, 2017). Relational needs have been strongly associated with improved health (vitality, self-determination, self-control, …) and wellbeing (Maslow, 1987; Baumeister and Leary, 1995; Kasser and Ryan, 1999; La Guardia et al., 2000; Ryan and Deci, 2000) and despite differences in theoretical approaches, there is a remarkable convergence among scientists on the fundamental importance of being connected to others (La Guardia and Patrick, 2008; Ryan and Deci, 2017).

Within the specific framework of integrative relational psychotherapy and transactional analysis, the relational needs model developed by Erskine (1996, 1997, 1998) is widely recognized (Pourová et al., 2020). This model emerged from a study of transference in psychotherapy and a qualitative investigation of the crucial factors in significant relationships conducted at the Institute for Integrative Psychotherapy in New York City in the early 1990s. However, relational needs are not only present in the context of psychotherapy; they are essential to a person’s sense of wellbeing throughout the life cycle (Erskine, 2015). In this respect, relational needs are the component parts of a universal human desire for the relationship; they are the *needs unique to interpersonal contact* (Erskine, 2015, p. 46). Relational needs are not the basic needs of life, such as food, air, or proper temperature, but they are the essential elements that enhance the quality of life and a sense of self-in-relationship. When a relational need is not satisfied, the need becomes more intense and is phenomenologically experienced as longing, emptiness, nagging loneliness, or an intense urge often accompanied by nervousness (Erskine et al., 1999). The continued absence of satisfaction with relational needs may be manifested as frustration, aggression, or anger. When disruptions in the relationship are prolonged, the lack of need satisfaction is manifested as a loss of energy or hope and shows up in *script beliefs*, such as “*No one is there for me*” (Erskine et al., 1999, Erskine and Moursund, 2011). These script beliefs are the cognitive defenses when needs do not get a satisfying response from another person (Erskine, 1980).

Žvelc et al. (2020) have emphasized this need for the relationship as a primary human motivation and have substantiated their perspective on several child development researchers and writers (Fairbairn, 1952; Bowlby, 1969, 1973, 1980; Kohut, 1971; Ainsworth et al., 1978; Stern, 1985; Fairbairn, 1986/1941; Winnicot, 1986/1960; Hesse, 1999). Attachment systems motivate infants to seek proximity and communication with caregivers. Therefore, attachment has an important evolutionary function—to heighten the possibility of survival of the child. Additionally, attachment relationship is crucial for the healthy development of both the brain and interpersonal relationships (Schore, 1994, 2001, 2003; Siegel, 1999; Cozolino, 2002). The qualities of affect and rhythmic attunement, relational needs, and sustained gestures of attachment between child and parent that provide the regulation of a child’s body sensations and affects are crucial for establishing a sense of safety, connection, and secure attachment (Erskine, 2021). In fact, attachment theory and research have underscored the importance of attachment and relational needs through the life cycle (Hazan and Shaver, 1987; Bartholomew and Horowitz, 1991; Hesse, 1999; Wallin, 2007).

The relational needs identified by Erskine and included in his model are the following (Erskine et al., 1999; Erskine, 2021): (1) *security* is the visceral experience of having our physical and emotional vulnerabilities protected. It involves the experience that our variety of needs and feelings are human and natural. Security is a sense of simultaneously being vulnerable and in harmony with another; (2) to *feel validated*, *affirmed*, and *significant* within a relationship requires the other person’s validation of the significance and function of our intrapsychic processes of affect, fantasy, and meaning-making, and to validate that our emotions are a significant intrapsychic and interpersonal communication. It includes the need to have all of our relational needs affirmed and accepted as natural. This need is a relational request for the other person to be involved by providing a quality of interpersonal contact that validates the legitimacy of relational needs, the significance of affect, and the function of intrapsychic processes; (3) *acceptance by a stable*, *dependable*, and *protective other person* is an essential relational need. Each of us as children had the need to look up to and rely on our parents, elders, teachers, and mentors. We need to have significant others from whom we gain protection, encouragement, and information. The relational need for acceptance by a consistent, reliable, and dependable other person is the search for protection and guidance; (4) the *confirmation of personal experience* is also an essential relational need. The need to have experience confirmed is manifested through the desire to be in the presence of someone who is similar, who understands because he or she has had a similar experience, and whose shared experience is confirmed. It is the quest for mutuality, a sense of walking, together with a companion who is “like me,” who has walked the same path in life. It is the need to have someone appreciate and value our experience because they phenomenologically know what that experience is like; (5) *self-definition is* the relational need to know and express one’s own uniqueness and to receive acknowledgment and acceptance from the other. Self-definition is the communication of one’s self-chosen identity through the expression of preferences, interests, and ideas without humiliation or rejection; (6) another essential relational need is *to have an impact on the other person*. Impact refers to having an influence that change the other in some desired way. An individual’s sense of competency in a relationship emerges from agency and efficacy, attracting the other’s attention and interest, influencing what may be of interest to the other person, and affecting a change in affect or behavior in the other; (7) the need to *have the other person initiate* refers to the impetus of another person’s making interpersonal contact. It is the reaching out to another, in some way that acknowledges and validates their investment in the relationship; and (8) the need to *express love* is an important component of relationships. Love is often expressed through quiet gratitude, thankfulness, giving affection, or doing something for the other person. The relational need to express love, whether it be from children to parents, sibling or teacher, or from a client to a therapist, is an important component in maintaining relationships. When the expression of love is stymied, the expression of self-in-relationship is thwarted.

Žvelc and Jovanoska (2016, 2017) and Žvelc et al. (2020) developed a new instrument for measuring the satisfaction of relational needs in the general population (RNSS). The final version is comprised of 20 items rated on a five-point Likert scale from agree to disagree. The scale yields five subscales and a total score of satisfaction of relational needs. The first four subscales (support and protection, have an impact, shared experience, and initiative from the others) reflect the relational needs described by Erskine. According to Žvelc et al. (2020), the fifth scale reflected the other four dimensions of the model as an overall dimension and was named need for authenticity. The instrument has been validated in Slovenian (Žvelc et al., 2020), Czech (Pourová et al., 2020), and Turkish (Toksoy et al., 2020) and showed good psychometric properties and the confirmation of the factorial structure and the hierarchical model.

Given the lack of instruments to measure relational needs in Spanish, the purpose of this article is to adapt the Relational Needs Satisfaction Scale (RNSS) using both a clinical and non-clinical sample and to analyze its psychometric properties. It is expected that the Spanish version of the RNSS presents adequate psychometric properties in terms of reliability and construct and discriminant validity, thus offering new evidence of the validity of the instrument.

## Materials and methods

### Design

**FIGURE 1:**
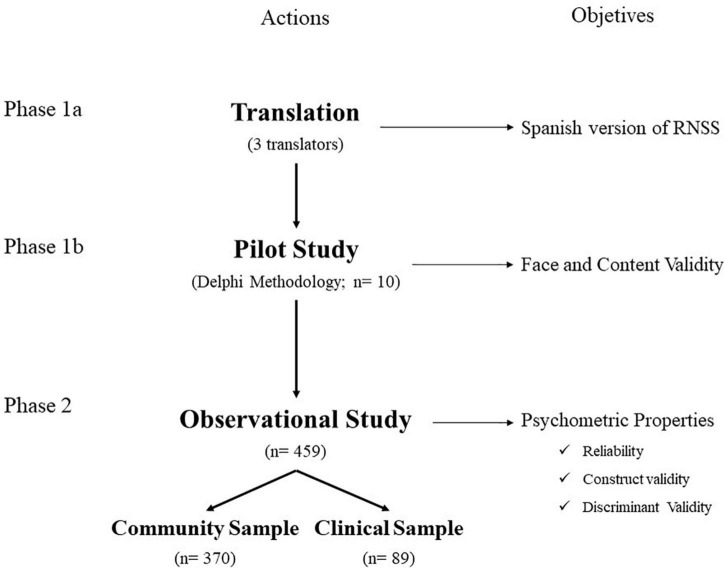
Flowchart of the methodological process.

It is an instrumental cross-sectional study to translate and adapt a psychometric instrument to Spanish following Sousa and Rojjanasrirat (2011) recommendations. The study was conducted between September 2018 and December 2019 in two different phases. In the first phase (Phase 1), which was conducted between September and December 2018, the process of translating and adapting the Relational Needs Satisfaction Scale from English to Spanish was undertaken. Whereas in the second phase (Phase 2), carried out between January 2019 and December 2019, the field study for the psychometric validation of the instrument was undertaken. Figure 1 shows the flow chart of the methodological process.

### Procedure

#### Phase 1.- translation of the RNSS (pilot study)

Acquadro et al. (2004) recommendations and methodology of translation were used. The first step consisted of the translation of the original version (in English) to the target version (in Spanish). In order to do this, three translators that had knowledge of both languages took part in the process. Two of them were native Spanish and one of them was native Irish. They translated the test independently. One was selected as the gold standard (T0), an expert on psychotherapy with formal education in integrative psychotherapy. The other two translators have formal graduate education in psychology (T1) and in international relationships (T2). Inter-rater agreement analysis was conducted taking as a reference the gold standard. Agreement and discrepancy with the standard were calculated utilizing inter-rater reliability with kappa coefficients (κ).

Once a Spanish version was produced, a pilot study with 10 volunteers was conducted following a Delphi methodology (Trevelyan and Robinson, 2015). The participants answered three questions to analyze the face and content validity. Using a 10-point response format, individuals were asked if (1) the items were worded in such a way that they were easily (0) or hardly (10) understood, (2) they were related (0) or they did not have relation at all (10) with the topic of relationships, and (3) if they felt represented (0) or not at all (10) with the proposed statements. The main aim of these questions was to be able to count with an indicator that could give information regarding the face and content validity of the RNSS.

#### Phase 2.- psychometric properties

To study the psychometric features of the translated scale, a cross-sectional observational design was used with a sample of volunteers that answered an informed consent that was included in the RNSS questionnaire.

### Sample and procedure

Four hundred fifty-nine participants were recruited. One sub-sample was a clinical group of 89 people being treated for emotional distress in a mental health center; the other, 370 participants from the community with no history of psychological problems. No statistically significant differences were found with respect to the socio-demographic characteristics of the two samples (age: *F* = 2.65, *p* = 0.104; sex: χ^2^ = 0.87, *p* = 0.207; employment status: χ^2^ = 2.23, *p* = 0.844). The mean age of the participants was 39.36 years (SD = 14.81) with ages ranging from 18 to 65 years; there was an equal distribution of men (51.2%) and women (48.8%), and the majority were employed (91%).

All participants were informed of the objectives of the study and were given the chance to clarify any doubts. Inclusion criteria were: being 18 years or older, volunteer participation, and absence of incapacitating conditions that could affect response patterns (cognitive impairment for reading and comprehension, severe mental health disorder, or substance abuse). The field study of the community sample was conducted by four trained persons with a background in psychology. Access to the sample was facilitated by the network of contacts with associations and institutions collaborating with the research team. The clinical sample was assessed by one of the researchers who carry out his clinical practice in a community mental health service. In this respect, in both cases, it is a non-probability opportunity sample. The study was approved by the research ethics committee and by those responsible for the organizations where their samples were obtained. Verbal consent was given by all participants and the anonymity and confidential treatment of the information provided were guaranteed.

### Instruments

#### Relational Needs Satisfaction Scale

Žvelc et al. (2020) the test has a total of 20 items and measures five conceptual dimensions of Erskine’s (2015) relational needs model allowing for five scalar scores (see section “Introduction”). Each of these scales consists of four items stated to be answered on a five-point Likert scale, from one (1) “*never true*” to five (5) “*always true*.” The original study obtained high reliability for the total scale (α = 0.90), as well as for the five dimensions (internal consistency values between 0.73 and 0.85). Convergent validity was also calculated with associations in the expected direction with life satisfaction and wellbeing.

#### Satisfaction with life scale

Diener et al. (1985) adapted by Vázquez et al. (2013) to Spanish. It consists of five items with a seven-point response format, from “*strongly agree*” to “*strongly disagree*.” It is a sound and widely used measure, and the internal consistency reported in the original study was high (α = 0.87), as well as the values reported in Spanish adaptation (α = 0.88). In our study, Cronbach’s alpha was 0.81.

#### Scales of psychological wellbeing

Ryff (1989) developed this instrument. In this study, the version proposed by Van Dierendonck (2004) and adapted to Spanish by Díaz et al. (2006) was used. The SPWB consists of 31 items rated on a six-point Likert scale, ranging from one (*totally disagree*) to six (*totally agree*), and allows the assessment of six dimensions of eudaimonic wellbeing. For the purposes of our study, we used a global measure of wellbeing based on the contribution of all items. The observed reliability of this global indicator was 0.91 for the present study.

### Statistical analysis

For the analysis of the RNSS items, the mean (M), standard deviation (SD), skewness (Sk), and the correlation coefficient between the items and the rest of the scale (r) were calculated, as well as the value of Cronbach’s alpha coefficient (α) if the item was removed.

The internal consistency of the total RNSS and its five dimensions was calculated for the total number of participants and for each of the samples (clinical vs. non-clinical), and we use Feldt’s test (Feldt, 1980) to test the hypothesis that the Cronbach’s alpha reliability coefficient is the same for two tests administered to the same sample. Based on the hypothesis that relational needs in emotionally disturbed people will be less satisfied than in non-emotionally disturbed people, we tested the differences in means between the two groups (clinical vs. non-clinical) using the ANOVA test.

To test the factor structure of the RNSS (Lorenzo-Seva and Ferrando, 2006), we checked the adequacy of the correlation matrix to ensure its factorization based on the Kaiser–Meyer–Olkin test and Bartlett’s sphericity test. Hull method (PA) (Lorenzo-Seva et al., 2011) and minimum average partial method (MAP) (Velicer, 1976) were carried out as extraction criteria for the advisable number of factors according to the configuration of the correlation matrix. Finally, the multivariate normality was analyzed with the Mardia (1970) test.

Subsequently, a confirmatory factor analysis (CFA) with covariance structural techniques using EQS (Bentler, 2004) was conducted. Maximum likelihood robust estimation was used to estimate the parameters. The chi-squared test (χ^2^) was used to evaluate the goodness of fit of the corresponding model and indicated that the probability that the variation between the sampling variance, covariance matrix, and the matrix resulting from the hypothesized model was random. In the event of non-compliance with the multivariate normality, estimations were calculated applying robust methods (Satorra and Bentler, 1990; Satorra, 2002). Given that the chi-square is sensitive to variations in sample size (Schermelleh-Engel et al., 2003), additional measurements of the goodness of fit of the model were used (Hu and Bentler, 1999), such as standardized root mean square residual (SRMR) and the root mean square error of approximation (RMSEA) and 90% confidence interval of RMSEA, which considers values <0.05 to be adequate and those <0.08 to be acceptable; the goodness-of-fit index (GFI), Bentler–Bonnet non-normal fit index (BB-NNFI), and comparative fit index (IFI), which considers values >0.90 to be adequate.

Three structure models were tested: a model of five correlated factors (F5C), a hierarchical model of five factors subsumed in a general second-order factor (F5F1), and a bi-factor model (Bi-F). The bi-factor model is a type of second-order confirmatory factor analysis that assumes the existence of a general factor that explains the covariance of all observed measures (Brown, 2014) and simultaneously presents several first-order factors that mediate and explain a portion of the same items (Chen et al., 2006). Thus, it is possible to specify the direct effects of the first-order factors and the general (second-order) factor, without necessarily being correlated. It is appropriate for measurement scales that provide an interpretable total score but have subdomains that have substantive value for research (Brown, 2014).

Finally, to test the convergent validity of the RNSS, we assessed the association of the RNSS total score and subscales with two reference instruments (life satisfaction, and Ryff’s scales of psychological wellbeing). Since some of the variables were non-normally distributed, we use the Spearman correlation coefficient.

## Results

### Phase 1. translation–back translation

Both translations from English to Spanish (T1 vs. T2) agreed on 18 items of the scale giving a level of agreement of 90%. Translator 1 (T1) coincided in 18 out of 20 items with the gold standard, and Translator 2 (T2) in 20. The degree of agreement in the inter-rater reliability, based on the Kappa coefficient, was high (κ = 0.87).

Table 1 presents the judgments of 10 volunteers who assessed the 20 items in terms of ease of understanding, relevance to relational needs, and appropriateness. In general, the scores are low, indicating that the items were easy to understand except for item 18, negatively worded. No item reflected an average score greater than five out of 10, not even in the maximum scores. The items that reflected a higher score are 2, 10, 16, and 18, with the highest mean being that of item 18 (*M* = 2.4) in the assessment of difficulty in understanding the item.

**TABLE 1:** Assessment of the understanding and adequacy of the items of the RNSS (*n* = 10).

		Easily (0) or Hardly (10) understood				Related (0) or not (10) with relations with people				Represented (0) or not (10) with the proposed statements			
Item	Wording	Min	Max	M	SD	Min	Max	M	SD	Min	Max	M	SD
01	My social circle consists of …	0	3	0.8	0.4	0	3	0.8	0.4	0	2	0.9	0.2
02	I hardly have to hide …	0	4	1.2	0.8	0	3	1.1	0.8	0	4	1.8	0.7
03	I have a strong, stable and …	0	2	0.7	0.3	0	1	0.2	0.3	0	3	0.9	0.6
04	I know a capable individual …	0	2	0.5	0.2	0	1	0.4	0.4	0	2	0.8	0.5
05	I know people who experience …	0	2	0.2	0.2	0	2	0.7	0.3	0	2	1.0	0.6
06	Others often take my advice …	0	3	0.9	0.3	0	2	1.1	0.4	0	3	1.2	0.8
07	Other people often help me …	0	2	0.7	0.3	0	2	0.4	0.4	0	2	0.4	0.6
08	I know people with a world- …	0	3	1.0	0.4	0	3	0.9	0.3	0	3	1.1	0.6
09	Other people sometimes …	0	2	0.8	0.2	0	2	0.8	0.4	0	2	0.9	0.6
10	People close to me would …	1	3	1.2	0.5	0	2	0.9	0.3	0	2	0.6	0.4
11	I feel free to show my …	0	2	0.9	0.4	0	1	0.3	0.2	0	1	0.5	0.3
12	I do not have to pretend …	1	3	1.1	0.6	0	2	0.6	0.5	0	2	0.9	0.4
13	I have at least one person in …	0	0	0.0	0.0	0	0	0.0	0.0	0	0	0.0	0.0
14	There are people in my life …	0	0	0.0	0.0	0	1	0.2	0.2	0	2	0.4	0.5
15	I feel that I have an influence …	0	0	0.0	0.0	0	1	0.3	0.3	0	0	0.0	0.0
16	I can show my true self …	0	3	1.2	0.5	0	2	0.9	0.5	0	2	0.9	0.5
17	In times of trouble, I have …	0	3	1.1	0.6	0	1	0.4	0.4	0	1	0.5	0.6
18*	No-one ever prepares.	2	6	2.4	0.9	2	4	1.7	1.2	2	4	2.1	1.2
19	I have noticed that other …	0	2	0.9	0.6	0	2	0.7	0.7	0	3	1.1	0.5
20	Other people often ask about …	0	1	0.3	0.2	0	1	0.4	0.2	0	2	0.8	0.4

Min, minimum; Max, maximum; M, mean; SD, standard deviation.

*, reverse items.

### Phase 2. psychometric characteristic

Table 2 presents the descriptive data of the 20 items that form the RNSS in the total sample. The percentage of individuals choosing each option, the mean, standard deviation, and skewness as statistics of central tendency and position are included. The table also includes the items reliability data, both for the subscales and for the total score. Finally, the last two columns present the results of the exploratory factor analysis, giving the communality values (h^2^) and factor loadings (λ) for each item.

**TABLE 2:** Descriptive and psychometric characteristics of RNSS (*n* = 459).

		Percentage distribution and descriptive statistics								Reliability				EFA	
										Subscales		Total Scale			
Item	Wording	1	2	3	4	5	M	SD	Sk	r	α	r	α	h^2^	λ
02	I hardly have to hide …	8.3	19.8	16.8	33.3	21.8	3.41	1.25	−0.38	0.380	0.708	0.270	0.873	0.196	0.442
11	I feel free to show my …	2.4	10.7	30.7	32.5	23.7	3.64	1.03	−0.36	0.522	0.616	0.576	0.866	0.457	0.676
12	I do not have to pretend …	8.9	6.8	12.9	31.2	40.3	3.87	1.26	−1.03	0.552	0.590	0.503	0.868	0.473	0.688
16	I can show my true self …	1.1	6.3	17.4	33.1	42.0	4.09	0.98	−0.88	0.516	0.624	0.627	0.864	0.444	0.666
	*Authenticity*						3.75	0.82	−0.35		0.699				
03	I have a strong, stable …	2.8	7.6	17.4	32.2	39.9	3.99	1.06	−0.92	0.640	0.775	0.466	0.869	0.523	0.723
04	I know a capable individ. …	0.7	6.3	12.0	32.9	48.1	4.22	0.93	−1.10	0.596	0.794	0.618	0.865	0.453	0.673
13	I have at least one person …	1.1	6.5	10.9	26.6	54.9	4.28	0.97	−1.29	0.705	0.746	0.548	0.867	0.667	0.816
17	In times of trouble. I …	4.4	9.8	24.6	29.6	31.6	3.74	1.13	−0.61	0.640	0.777	0.525	0.867	0.518	0.720
	*Support and Protection*						4.05	0.82	−0.88		0.820				
06	Others often take my …	9.4	33.6	32.5	18.7	5.9	2.78	1.04	0.26	0.409	0.765	0.276	0.876	0.215	0.464
15	I feel that I have an influence …	8.7	29.2	36.5	20.9	4.6	2.83	1.00	0.09	0.577	0.668	0.429	0.871	0.466	0.683
19	I have noticed that other …	3.5	22.4	38.8	29.8	5.4	3.11	0.93	−0.08	0.630	0.641	0.523	0.863	0.624	0.790
20	Other people often ask.	2.6	18.3	40.1	31.2	7.8	3.23	0.92	−0.08	0.568	0.675	0.497	0.868	0.492	0.702
	*Having an Impact*						2.99	0.73	0.01		0.747				
01	My social circle consists …	5.4	18.3	26.1	38.8	11.3	3.32	1.06	−0.37	0.446	0.699	0.443	0.870	0.285	0.534
05	I know people who …	2.4	14.8	34.6	35.5	16.5	3.41	0.96	−0.22	0.385	0.727	0.537	0.867	0.207	0.455
08	I know people with a …	2.6	14.6	292	36.2	17.4	3.51	1.02	−0.32	0.602	0.601	0.626	0.864	0.574	0.758
14	There are people in my …	0.9	9.6	27.5	36.8	25.3	3.76	0.96	−0.38	0.619	0.575	0.638	0.864	0.617	0.785
	*Shared Experience*						3.53	0.79	−0.26		0.721				
07	Other people often help …	5.4	27.0	34.4	25.7	7.4	3.03	1.02	0.05	0.373	0.312	0.454	0.870	0.453	0.653
09	Other people sometimes …	1.3	10.5	29.0	40.1	19.2	3.65	0.94	−0.37	0.312	0.378	0.529	0.867	0.192	0.434
10	People close to me would …	2.6	19.8	35.5	31.8	10.2	3.27	0.97	−0.07	0.452	0.236	0.519	0.868	0.581	0.756
18*	No-one ever prepares …	2.2	8.7	24.6	33.1	31.4	3.83	1.03	−0.59	0.028	0.631	0.049	0.883	0.041	0.037
	*Initiative from Other*						3.44	0.62	0.11		0.480				
	*RNSS Total Score*						3.54	0.56	−0.43				0.875		

M, mean; SD, standard deviation; Sk, skewness; r, correlation coefficient of the item with the rest of the scale/subscale; α, Cronbach’s alpha: value of the reliability if the items is removed; EFA, exploratory factor analysis; h^2^, communality; λ, factorial weights. *, reverse item.

As can be seen in the table, the mean of the total scale is 3.54, in a range from 1 to 5 points. All the items, except items 06, 07, and 15, show a smooth negative asymmetry which means that people tended to answer with high scores. There is one item where the asymmetry is presented in an important way (out of rank: Sk > 1.25), this happens in item 13 with an asymmetry of −1.29. In general, it is possible to see the ceiling effect in two items (12 and 16) of the authenticity subscale, and in two items (04 and 13) in protection. Thus, in these two dimensions, there are items that more than 40% of the participants responded to option 5. This effect does not happen in the other dimensions of the RNSS. No floor effects (low percentages in more than 40% of the answers) were observed.

The reliability of the total of items of the RNSS was high (α = 0.875), and the withdrawal of any of its items would not allow obtaining a notorious increase in its internal consistency. Two subscales showed high alpha values (protection α = 0.82 and having impact α = 0.75), two other subscales had acceptable values (authenticity α = 0.70 and shared experience α = 0.72), and finally, the initiative from the other subscale had a low internal consistency value (α = 0.48). Note that the removal of item 18 would increase the reliability of the initiative dimension to 0.63. In parallel, the communalities are low (h^2^ < 0.20) in three items (02, 09, and 18), although only item 18 shows an insufficient factor loading (λ < 0.40).

Table 3 presents the means and standard deviations for the five subscales and the total scale, for the two samples, along with the reliability values of the scales in each sub-sample. The analysis of variance shows statistically significant differences for all contrasts except for the dimensions “initiative from others” (*F* = 0.92; *p* = 0.323) and “having an Impact” (*F* = 2.33; *p* = 0.127), with higher mean values in the community sample, indicating a higher satisfaction of relational needs than the clinical sample. However, the effect sizes achieved are moderate to low (*d* < 0.40).

**TABLE 3:** Descriptive statistics and reliability for each sub-sample, intergroup contrast, and correlations between RNSS scales for total sample (*n* = 459).

		Community group (*n* = 370)			Clinic group (*n* = 89)			ANOVA and effect sizes				Correlation for total sample				
		M	SD	Alpha	M	SD	Alpha	F	p	η^2^	d	1	2	3	4	5
1	Authenticity	3.81	0.81	0.718	3.48	0.83	0.590	11.65	<0.001	0.026	0.33					
2	Support and Protection	4.10	0.81	0.826	3.86	0.88	0.791	5.66	0.018	0.012	0.22	0.45				
3	Having an Impact	3.01	0.71	0.750	2.38	0.82	0.734	2.33	0.127	0.005	0.14	0.35	0.25			
4	Shared Experience	3.55	0.72	0.721	3.28	0.79	0.698	9.54	0.002	0.021	0.29	0.55	0.54	0.52		
5	Initiative from Other	3.46	0.60	0.457	3.38	0.69	0.537	0.92	0.323	0.002	0.09	0.45	0.47	0.36	0.52	
6	Total Score	3.59	0.54	0.874	3.38	0.62	0.878	8.35	0.005	0.021	0.29	0.76	0.74	0.65	0.84	0.73

M, mean; SD, standard deviation; α, Cronbach’s alpha: value of the reliability; F, ANOVA test; p: probability value; η^2^, partial eta square; d, Cohen’s d coefficient.

The internal consistency of the total scale in both samples is the same (α = 0.87), and equivalent to the rest except in the case of the authenticity subscale where it is significantly higher (Feldt’s test = 0.68, *p* = 0.008) in the community sample (α = 0.72) than in the clinical sample (α = 0.59). Regarding the association between the subscales of the RNSS (right section of Table 3), positive and statistically significant correlations (*p* < 0.01) are observed between all dimensions with values ranging from *r* = 0.25 (protection with having an impact) to *r* = 0.55 (shared experience with authenticity). The correlations of each dimension with the total scale are high in all cases (*r* ≥ 0.66).

Correlations with life satisfaction and psychological wellbeing were positive and statistically significant (authenticity: 0.17 and 0.40, protection: 0.22 and 0.26, having an impact: 0.10 and 0.28, shared experience: 0.17 and 0.36, initiative from other: 0.09 and 0.24, total score: 0.20 and 0.42), although the correlation values have been moderate.

To assess whether the data could be subjected to factor analysis, the correlation matrix was tested. The Kaiser–Meyer–Olkin (KMO = 0.916) and Bartlett sphericity tests [χ^2^_(190)_ = 3211.2; *p* < 0.001] showed that the items of the RNSS have intercorrelations and, therefore, the matrix is susceptible to be factored. Likewise, the Velicer (MAP) and Hull method suggest that a major factor should be retained. The exploration of the possible resulting factors by means of an exploratory strategy through the FACTOR program offers a solution of four factors with eigenvalues superior to 1. However, the first factor offers an eigenvalue of 6.83, compared with an eigenvalue of 1.57 for the second factor. Since the first factor is more than three times higher than the second factor, the presence of a major factor is assumed and the residual factors are disregarded (Gorsuch, 1983).

The result of the factorial exploration of the correlation matrix suggests the presence of the main factor in our data sample. For this reason, a structure model of RNSS has been tested, assuming that the items of the instrument allow for differentiating five dimensions and that these, in turn, conform to a second-order main latent factor referred to as relational needs. Alternatively, the existence of a bi-factor model has also been tested where RNSS items saturate simultaneously on a general factor and its five corresponding dimensions. A five-factor correlated model has also been tested. To test this model, the correlation matrix was subjected to a confirmatory factorial analysis through the EQS program. Since the correlation matrix presents multivariate asymmetry (Mardia index = 45.12), the maximum likelihood robust method was used to estimate the models. These models have been tested for the total sample (*n* = 459), and the results are shown in Table 4.

**TABLE 4:** Confirmatory factor analysis of RNSS for the total sample. Fit indices are estimated using the robust method.

Sample	Model	χ^2^	df	χ^2^/df	AIC	GFI	BB-NNFI	CFI	SRMR	RMSEA	(90% CI)		Rho
Total (*n* = 459)	F5C	405.41	160	2.53	85.42	0.901	0.900	0.907	0.058	0.058	(0.051	0.065)	0.900
	F5F1	429.63	165	2.60	99.63	0.897	0.885	0.900	0.069	0.059	(0.052	0.066)	0.900
	Bi-F	333.15	150	2.22	33.15	0.918	0.912	0.931	0.053	0.052	(0.044	0.059)	0.909

Model: F5C: five-factor correlated; F5F1: hierarchical model (SOFCA): Bi-F: Bi-factor model. Method: NDT: normal distribution theory; Robust: robust method.

Fit Indices, χ^2^: Chi-square; df, degree of freedom; AIC, Akaike’s information criterion; GFI, goodness fit index; BB-NNFI, bentler–bonnet non-normed fit index; CFI, comparative fit index; SRMR, standardized root mean square; RMSEA, root mean square error of approximation; CI, confidence interval; Rho, reliability coefficient.

**FIGURE 2:**
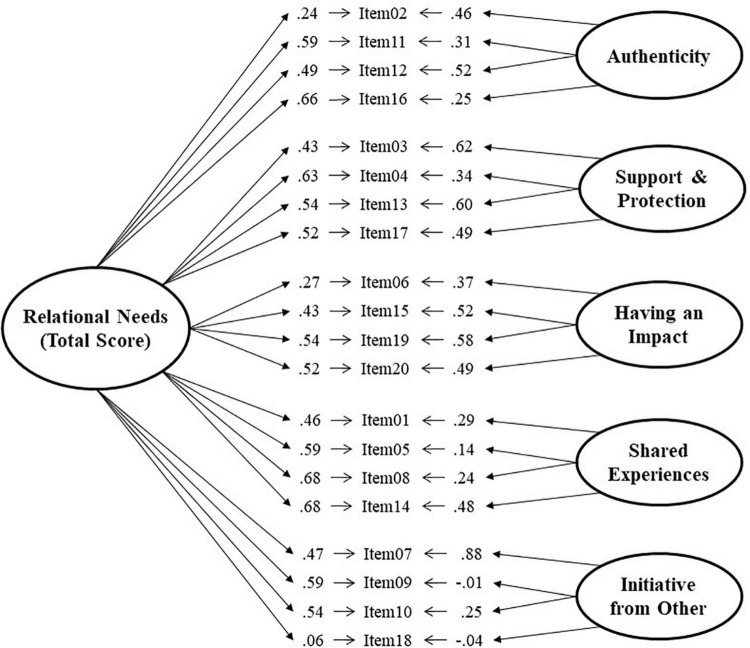
Bi-factor model of RNSS.

**FIGURE 3:**
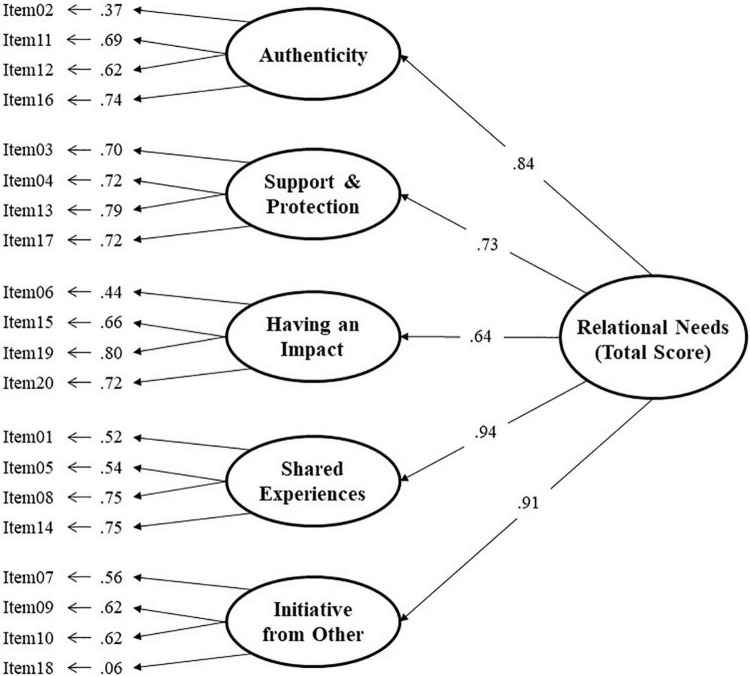
Hierarchical model of RNSS.

The three models show adequate fit indices, but the model with the best fit is the bi-factor model [χ^2^_(150)_ = 333.15; *p* < 0.001; GFI = 0.92, BB-NNFI = 0.91, CFI = 0.93, RMSEA = 0.052, 90% IC = 0.044 to 0.059] over the other two alternative models. The graphical representation and factor loadings of the bi-factor model for the total sample are shown in Figure 2, and the SOCFA model in Figure 3.

In general, the items tend to have higher loadings on the general factor, with only three items having low loadings of less than 0.35 (λ_02_1_ = 0.24, λ_06_1_ = 0.27, and λ_18_1_ = 0.06). The items that present a lower saturation in the general factor, present a higher weight in the dimensional factor [item 02 in “authenticity” (λ_02_2_ = 0.46), and item 06 in “having an impact” (λ_06_3_ = 0.37)], except for item 18 which presents low saturations in both the general factor (λ_18_1_ = 0.06) and the dimensional factor (λ_18_6_ = −0.04). Item 9 does not contribute to the dimension “initiative from other” (λ_09_6_ = −0.01), but showed an acceptable load (λ_09_1_ = 0.59) in the general factor.

The solution of the hierarchical or SOCFA model (Figure 3) shows factor loadings above 0.40 for all items, except for item 18 with loading of almost zero. Factor saturations on the second-order factor are above 0.70. These results are very similar to those obtained in the three international studies previously carried out (Pourová et al., 2020; Toksoy et al., 2020; Žvelc et al., 2020).

## Discussion

The main objective of this study was to adapt to Spanish a scale that allows the measurement of relational needs for subsequent use in clinical practice contexts and in research with non-clinical populations. The observed results allow us to conclude that the Spanish adaptation of the RNSS offers adequate characteristics of language adaptation and measurement.

Inter-rater agreement of the translated version was appropriate. Likewise, the assessment made by 10 people of the characteristics of the item statements allows us to accept that they are easy to understand, that the items are related to the construct they intend to measure, and that the items are in the repertoire of interpersonal relationships (see Supplementary Table 1 for the final version of the Spanish scale). However, in the pilot study, it was already possible to identify some items that showed a differential behavior. Of these, item 18 is the item with the greatest number of problems in the psychometric adequacy of the scale. Nevertheless, we believe that the results obtained in the pilot study provide evidence for the face and content validity of the RNSS and that the translation is appropriate.

In general, the metric behavior of the items is acceptable. Mostly negative asymmetries are observed, indicating a tendency on the part of the participants to report higher relational satisfaction scores. This effect is more marked in the items belonging to the “protection” dimension (items 04 and 13), but there are no outliers in any of the remaining items. In this respect, item response behavior follows a pattern similar to that observed in other instruments used in psychological assessment (Holgado-Tello, 2015).

With respect to the internal consistency of the items, item 18, integrated into the dimension of “initiative from others,” performed poorly. This item is the only one stated negatively and, as mentioned previously, it was one of the items that presented the greatest difficulties of comprehension. It is very likely that its different wording produces a response bias that could perhaps be corrected by changing its wording to positive.

Independently of the effect observed in item 18, the internal consistency shown for the total scale was high (α = 0.87), very similar to that obtained in the original study (Slovenia, α = 0.90), and in the two countries that adapted it to their language (the Czechia, α = 0.90; and Turkey, α = 0.83). However, if we look at the reliability observed in the dimensions, we see that performance is lower in the present study than in the Slovenian or Czech studies (Pourová et al., 2020; Žvelc et al., 2020), and more similar to the results obtained in the Turkish study (Toksoy et al., 2020).

In our study, the internal consistency observed in the community and clinical sample was equivalent except for the authenticity dimension. It has also been observed that, with the exception of the dimensions initiative from others and having an impact, the clinical sample had a statistically significant lower perception of satisfaction of needs in their relationships. This result allows us to accept that the RNSS shows evidence of discriminant validity.

Another important aim of our study was to confirm the factor structure of the RNSS. We have found empirical support that allows us to accept both the hierarchical structure of five factors subsumed in an overall factor proposed in the original study (Žvelc et al., 2020) and in the two adaptation studies (Pourová et al., 2020; Toksoy et al., 2020), as well as the proposed bi-factor model. Of the two, we favor the bi-factor (Bi-F) model based on the following criteria. Several authors (Reise et al., 2010, 2013; Chen et al., 2013) have summarized the advantages of the Bi-F model over second-order confirmatory factor analysis (SOCFA). The main advantage is that the Bi-F analysis allows to directly observe to what extent an item or scale (the observed variable) reflects a common target construct (i.e., a general factor) and, simultaneously, to what extent it may reflect a (domain-specific) sub-dimension. Consequently, the Bi-F model allows for the retention of a single common latent factor but also controls for variance arising due to additional common factors.

A second advantage of the Bi-F model is that in the SOCFA model it is not possible to observe direct relationships between the observed variables and the general factor, but rather an “indirect effect” or a “mediated relationship” through the first-order factors. Therefore, to estimate the variance attributable to the general factor, the loading of the observed variable on the domain-specific factor must be multiplied by the loading of the domain-specific factor on the general factor (Chen et al., 2006, 2013). In contrast, the Bi-F model provides information on all factor loadings and makes it possible to identify whether a domain-specific factor uniquely contributes to the prediction of external criteria (Chen et al., 2006, 2013). Given that in a Bi-F model the general and domain-specific factors are orthogonal, even a simple inspection of the item loadings on the second- and first-order factors is informative.

Since the SOCFA model is nested in the Bi-F model, no such restrictions are present. The latter can be used as a base model to compare model fit as the model becomes more constrained, which is a third advantage of the Bi-F model over SOCFA (Brown, 2014). For example, in a SOCFA model, correlations between first-order factors are assumed to occur because they have a common cause (i.e., the overall factor). Therefore, the observation of low loadings on a domain-specific factor and high loadings of the specific factors on a general factor may suggest that these variables are best explained by a general factor and do not constitute a domain-specific factor. Thus, if the items mainly reflect the general factor and have low loadings on the first-order factors, the sub-scales make little sense.

Our results with the SOCFA/hierarchical model are very similar to those obtained in the previous studies (Pourová et al., 2020; Toksoy et al., 2020; Žvelc et al., 2020) and, as the researchers have concluded, the five-dimensional model that would represent Erskine’s theory and the option of a general factor that would allude to the concept of relational needs would be satisfied. However, the Bi-factor model offers other possible conclusions. The data collected in the Spanish sample preferably offered the option of a general factor. The Velicer analysis and the solution of the Bi-factor model seem to favor this option. The factor loadings are of larger effect size than in the specific dimensions, although this does not rule out the contribution of the items to the latent factors representing the dimensions explored. Further work is needed to confirm this solution, and one possibility would be to seek an integration of all the data obtained in the various studies that have examined the evidence for the validity of the RNSS.

In sum, the adaptation to Spanish of the RNSS has shown an appropriate inter-rater agreement in the translation of the Spanish statements, as well as in the functionality of the item comprehension and the use of the relational needs valuation. In other words, the scale shows face and content validity. Besides, the results demonstrated that the RNSS has an adequate accuracy of measurement with sufficiently high coefficients and appropriate construct validity that is expressed through a suitable index of adjustment on the confirmatory factorial analysis. Therefore, this study gathers evidence to consider the translated RNSS as an appropriate instrument for the measurement of the mentioned construct. Nevertheless, it is necessary to adjust some aspects of the formulation of an item (number 18) and the execution of new studies with more controlled designs to demonstrate other evidence of validity. It will be advisable to check the invariance of the dimensional structure of the RNSS in different populations (clinical vs. community samples) or in different cultures and relational contexts (individualistic vs. Collectivistic cultures). Furthermore, we could also test the discriminative power of the RNSS in these conditions and the predictive validity as it regards functionality, health, and wellbeing. Finally, the instrument has been developed after Erskine’s theory of relational needs, and thus it can be a useful tool within the framework of integrative psychotherapy, both for diagnostic and assessment purposes.

## Data availability statement

The raw data supporting the conclusions of this article will be made available by the authors, without undue reservation.

## Ethics statement

The studies involving human participants were reviewed and approved by the Ethical Committee of the University of Deusto (ETK-19/19-20). The patients/participants provided their written informed consent to participate in this study.

## Author contributions

II designed the study, articulated the methodology and results in sections of the manuscript, conducted the data analysis, and wrote the draft manuscript. IG-M, AM, and SM-R played a role in item development and data collection. RE supervised the research and contributed to the writing of the manuscript. SG was responsible for writing the manuscript and contributed to the discussion of the results. GŽ contributed to the design of the study and the discussion. All authors reviewed and approved the final version of the manuscript.
