# Supplementary material for: Adaptation to Spanish of the “Relational Needs Satisfaction Scale”: Translation and psychometric testing
Source: Front Psychol. 2022 Aug 23;13:992205. doi: 10.3389/fpsyg.2022.992205 (PMC9445878; doi:10.3389/fpsyg.2022.992205)
Supplement: Supplementary file 1 [file Table_1.PDF]

Annex 1.- Translation of the Relational Needs Scale

	English	Spanish
1	My social circle consists of people who share a similar life experience (e.g., a hobby, a profession, belonging to the same group or online forum)	Mi círculo social consiste en personas que han compartido experiencias de la vida similares a las mías (por ejemplo: aficiones, profesión, ser parte del mismo grupo o foro online)
2	I hardly have to hide anything in the company of people close to me	Casi nunca tengo que esconder nada cuando estoy en compañía de personas cercanas
3	I have a strong, stable and protective person in my life, whom I can rely on	Tengo una persona fuerte, estable y protectora en mi vida que puedo contar
4	I have a capable individual who would help me if I found myself in trouble	Sé de alguna persona que me ayudaría en el caso de estar en peligro
5	I know people who experience some things similarly to me	Se de algunas personas que han pasado por las mismas experiencias que yo
6	Other often take my advice to heart	Hay personas que se toman mis opiniones a pecho
7	Other people often help me even if I do not specifically ask them to	La gente frecuentemente me ayuda sin que yo les pida ayuda
8	I know people with a Word-view similar to mine	Conozco a personas que tienen la misma forma de ver la vida que yo
9	Other people sometimes surprise me in a nice way	A veces hay personas que me sorprenden positivamente/ agradablemente
10	I see that other people listen to my advice or my suggestion	Veo que la gente escucha mis consejos y sugerencias
11	People close to me would sometimes do things for me without me having to ask	A veces la gente que es cercana a mí hace cosas para mí sin que yo les pida nada
12	I feel free to show my feelings to others and speak my mind, because I know they accept me for who I am	Me siento libre de expresar mis sentimientos y decir lo que pienso a los demás porque se que me aceptan tal como soy
13	I do not have to pretend with people who are important to me	No tengo que aparentar con personas que son importantes para mí.
14	I have at least one person in my life who encourages me, protects me or provides me with the information I need	Tengo al menos una persona en mi vida que me anima, protege y/o proporciona la información que necesito
15	There are people in my life with whom I share similar experiences	Hay personas en mi vida con quienes comparto experiencias similares
16	I can show my true self to people who are important to me without fear or rejection	Puedo enseñar mi verdadero yo a las personas que son importantes para mi sin miedo o rechazo
17	In times of trouble, I have someone who stands by me and who is strong enough to handle my problems	Cuando hay problemas, tengo alguien que me apoya y que es suficientemente fuerte para encargarse de mis problemas
18*	No-one ever prepares a nice surprise for me	Nadie prepara ninguna sorpresa agradable para mí
19	People encourages me to follow my own judgement regardless of their wishes.	La gente me anima a seguir mi propio juicio sin tener en cuenta sus deseos
20	Other people often ask about my opinion on a certain topic	Frecuentemente la gente me pregunta por mi opinión en ciertos temas
